# Plasmoacanthoma*

**DOI:** 10.1590/abd1806-4841.20164673

**Published:** 2016

**Authors:** Bruna Backsmann Braga, Alexandre Ozores Michalany, Jayme de Oliveira Filho, Luiz Carlos Cucé

**Affiliations:** 1 Clínica privada – São Paulo (SP), Brazil; 2 Universidade de Santo Amaro (UNISA) – Santo Amaro (SP), Brazil; 3 Hospital do Servidor Público Municipal (HSPM) – São Paulo (SP), Brazil

**Keywords:** Cheilitis, Cell proliferation, Mouth mucosa, Plasma cells

## Abstract

Plasmoacanthoma is an extremely rare verrucous tumor located on periorificial regions characterized by dense dermal plasmacytic infiltrates. Some authors classify it as a form of reactive plasma cell proliferation which represents a heterogeneous spectrum of mucocutaneous disorders. These plasma cell proliferations have been considered to be a benign immunologic inflammatory reaction to known or unknown stimuli. However, the etiology of plasmoacanthoma remains highly speculative. We report the case of a 40-year-old woman who presented with a lobulated warty lesion affecting the lower lip. Biopsy from the lesion was compatible with plasmoacanthoma, which remains an underreported disease in the dermatology literature.

## INTRODUCTION

Plasmoacanthoma is an extremely rare verrucous tumour located on periorificial regions characterized by dense dermal plasmacytic infiltrates with pronounced psoriasiform changes in the epidermis. Some authors classify it as a form of reactive plasma cell proliferation that represents a heterogeneous spectrum of cutaneous and mucocutaneous disorders.^1,2^

These plasma cell proliferations have been considered to be a benign immunologic inflammatory reaction to known (infection, friction, trauma, etc.) or unknown stimuli. However, the etiology of plasmoacanthoma remains highly speculative.^2^ In 1952 Zoon originally described dense plasma cell infiltrates occurring on the glans penis. Since then the disease has been reported under a wide variety of names depending on the involved mucosa (penis, vulva, perineum, lips, buccal mucosa, palate, gingivae, tongue, epiglottis or larynx). ^3^ In 1986 White *et al.* classified this group of similar disorders involving different body parts under the nomenclature "plasma cell mucositis."^4^ After that, however, some cases of plasma cell proliferations located exclusively on the skin have been reported.^1^

## CASE REPORT

**Figure 1:**
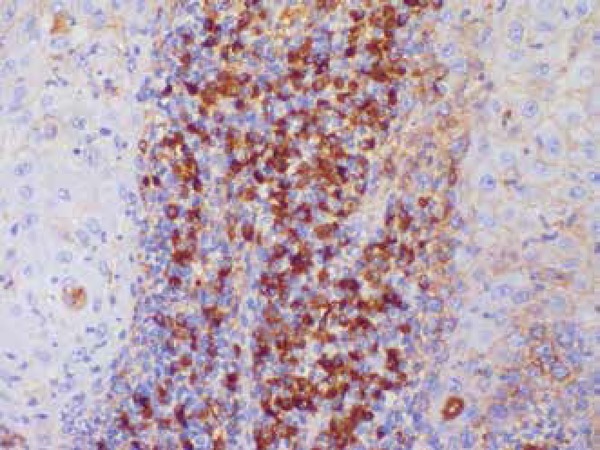
Lobulated warty lesion affecting lower lip. (frontal aspect)

We report a 40-year-old woman who presented with a lobulated warty lesion affecting the lower lip (Figure 1). It first appeared over 7 years earlier during her first pregnancy, but disappeared spontaneously. Five years later, during her second pregnancy, the lesions reoccurred and have developed since then. The patient sought dental care service that performed a series of exams, including biopsy. Results were all inconclusive.

**Figure 2:**
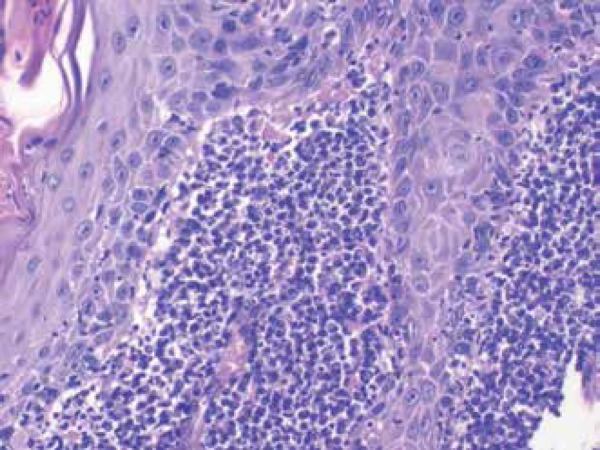
Dense predominantly plasmocytic infiltrate throughout the entire superficial dermis.

**Figure 3:**
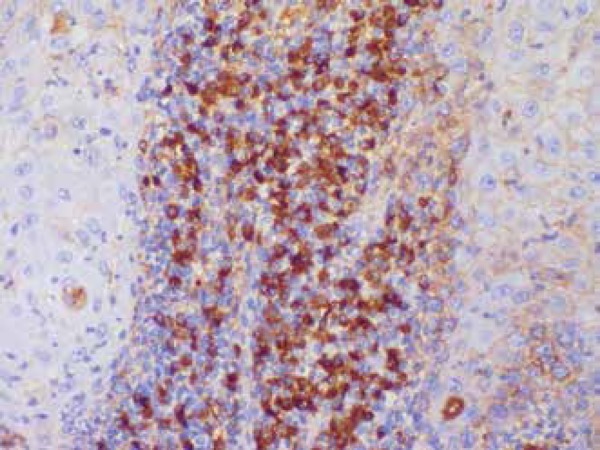
Immunohistochemistry showing Kappa positivity

Considering the clinical hypothesis of leishmaniasis, syphilis or squamous cell carcinoma, we performed complete blood work, venereal disease research laboratory (VDRL), FTA-ABS, Montenegro's intradermal reaction tests and an incisional biopsy. Histopathologic examination revealed a dense predominantly plasmocytic infiltrate throughout the entire superficial dermis. The epidermis presented thickening and elongation of the rete ridges – which correspond to the clinical features of the disease – along with the presence of eosionophilic bodies and exocytosis of lymphocytes (Figure 2). We observed no signs of neoplasia or malignancy. The search for fungal organisms and parasites through Periodic Acid Schiff Stain (PAS) was negative. Blood tests were normal and a Montenegro skin test was negative. After a review of the literature and given the clinical and histologic evaluation, we diagnosed plasmoacanthoma. An immunohistochemestry study showed positivity for kappa, lambda and CD 138, confirming a polyclonal infiltration in inflammatory reaction (Figure 3). We proceeded with three consecutive monthly 1ml-doses of triamcinolone acetonide injections (20mg/ml). The lesions gradually improved with no recurrence in the past 6 months.

## DISCUSSION

Mucocutaneous plasma cell proliferations represent a heterogeneous and rare group of dermatologic disorders.^1^ Plasmoacanthoma is a verrucous tumor involving the oral mucosa, particularly oral commissures. Perianal, periumbilical, inguinal area and toe web involvement have also been reported.^2^ Plasma cell cheilitis is also a rare inflammatory disorder that shows a characteristic dense infiltrate of plasma cells in the upper dermis.^3^ The usual clinical feature of plasma cell cheilitis is analogous to Zoon's plasma cell balanitis, aside from the characteristic affected area. In plasma cell cheilitis we observe an asymptomatic patch or plaque of erythema and induration of the lower lip in an elderly person^2^

The etiology of these benign plasma cell infiltrations is still obscure.^1^ Reports on similar rare inflammatory conditions with marked plasma cell infiltration advocate a hypersensitivity reaction to certain allergens.^5^ In plasma cell cheilitis, for example, the role of T cells and macrophages in B cell growth and differentiation has been reported. Studies suggest that similar mechanisms are involved in both the mucosa and cutaneous disorders.^3^ However, further investigation is required.

In both entities – plasmoacanthoma and plasma cell cheilitis – the significance of trauma and/or chronic irritation has been emphasized due to an enhanced incidence in persons habitually chewing tobacco and certain gums, using dentifrices or artificial dentures.^2^ Despite the relevant occurrence during pregnancies, we could not find any causative agent in our case. The patient denied any of the conditions cited above, and her orodental hygiene was attested by dental evaluation.

Topical, intralesional, and systemic corticosteroids, antibiotics, griseofulvin, etretinate, cyclosporin, excision or destructive procedures (Co2 laser ablation, electrocoagulation, cryosurgery) and radiation therapy have all been applied as therapeutic modalities with inconsistent success.^1,6-8^ Consistent with other reports, our patient responded to intralesional corticosteroids. However, due to emotional distress caused by the lesions, she remains under monthly follow up as well as an interdisciplinary approach with psychologists and a dental team.

Many terms have been used to describe the various clinical manifestations of idiopathic plasmacytic infiltrations. We agree that there is a discrepancy in the nomenclature, making the diagnosis challenging. Plasmoacanthoma remains an underreported disease in the dermatology literature, which makes it difficult to clearly understand its pathogeneses and consequently to indicate the best form of therapy. In conclusion, the long-term prognosis of patients with benign plasma cell proliferation is good, considering the absence of reports on progression to malignancy.^1^
